# Radiographic Texture Analysis of Densitometric Calcaneal Images: Relationship to Clinical Characteristics and to Bone Fragility

**DOI:** 10.1359/jbmr.090714

**Published:** 2009-07-13

**Authors:** Tamara Vokes, Diane Lauderdale, Siu-Ling Ma, Mike Chinander, Keona Childs, Maryellen Giger

**Affiliations:** 1 Departments of Medicine, The University of Chicago Chicago, IL, USA; 2 Health Studies, The University of Chicago Chicago, IL, USA; 3 Radiology, The University of Chicago Chicago, IL, USA

**Keywords:** noninvasive assessment, bone structure, vertebral fractures, densitometry, fracture risk

## Abstract

Osteoporotic fractures are related not only to bone mineral density (BMD) but also to bone structure or microarchitecture, which is not assessed routinely with currently available methods. We have developed radiographic texture analysis (RTA) for calcaneal images from a peripheral densitometer as an easy, noninvasive method for assessing bone structure. We conducted a cross-sectional study of the relationship between RTA and prevalent vertebral fractures (*n* = 148) among 900 subjects (ages 19 to 99 years, 94 males) referred for bone densitometry as part of their routine medical care. RTA features were derived from Fourier-based image analysis of the radiographic texture pattern (including root mean square, first moment, and power spectral analyses). RTA features were associated with age, weight, gender, and race, as well as glucocorticoid use. When controlling for clinical risk factors and BMD (or a summary measure calculated using FRAX algorithms), RTA features were significantly different for subjects with and without prevalent vertebral fractures [adjusted odds ratio (OR) = 1.5 per 1 standard deviation (SD) decrease in RTA feature beta, 95% confidence interval (CI) 1.2–1.8, *p* = .001]. Gender and use of pharmacologic therapy for osteoporosis did not significantly affect this association, suggesting that RTA can be applied to a wide range of densitometry patients. We conclude that RTA obtained using a portable instrument has a potential as a noninvasive method to enhance identification of patients at increased risk of osteoporotic fractures. Copyright © 2010 American Society for Bone and Mineral Research

## Introduction

The risk of osteoporotic fractures is usually assessed by measuring bone mineral density (BMD) using dual-energy X-ray absorptiometry (DXA). BMD is a good predictor of fractures in population studies(1) but performs less well in individual patients, as evidenced by a large overlap in BMD values between patients with and without fractures.(2–4) This is so because bone strength is determined not just by bone mass but also by bone quality or structure, which cannot be assessed easily by currently available methods. Three-dimensional imaging techniques for assessment of bone microarchitecture such as micro-CT and MRI are promising (reviewed in ref. (5)) but not practical for widespread clinical use owing to their high cost and/or radiation exposure and limited availability.

Bone architecture also can be assessed on radiographs. Several groups have shown that computerized radiographic texture analysis (RTA) performed on digitized radiographs differentiated patients with and without fractures when applied to spine,(6,7) proximal femur,(8) or calcaneus.(9,10) Further, RTA-derived indices correlated with histomorphometric findings(11) and with biomechanical properties of ex vivo specimens.(12,13) Finally, fractal analysis differentiated sportswomen with and without stress fractures, although the two groups had similar activity level, age, body mass index (BMI), BMD, and heel ultrasound measurements.(14)

We have applied the RTA methods that have been used previously on radiographs to calcaneal images that were obtained using a portable densitometer specially equipped with a high-resolution camera. Such densitometric images of the calcaneus had physical image quality suitable for RTA(15) and adequate short- and long-term precision of RTA.(16) In addition, there was a reasonably good correlation between RTAs from densitometric and radiographic images.(17) We have reported previously that among 170 postmenopausal women with no secondary causes of and no pharmacologic therapy for osteoporosis, RTAs of densitometric calcaneal images differentiated patients with and without prevalent vertebral fractures.(18) We now present findings obtained in a large sample of subjects recruited during their routine clinical BMD testing. In addition to demonstrating that RTA differentiated subjects with and without vertebral fractures in a more heterogeneous population, we also define the relationship between densitometric RTA and anthropomorphic variables and clinical risk factors and their summary measure derived using the FRAX algorithm that have not been reported to date.

## Methods

### Study subjects

This was a convenience sample that included 1075 ambulatory subjects (953 women) recruited when they presented for BMD measurement as part of their clinical care between 2001 and 2008. The study was approved by the University of Chicago's Institutional Review Board, and all participants signed a written informed consent. The densitometry facility performs all BMD testing at the University of Chicago; patients are referred primarily by University of Chicago physicians. Primary-care patients come generally from the surrounding communities, whereas tertiary-care patients may come from the greater metropolitan area or even the tristate region. It is not known whether study subjects were primary- or tertiary-care patients because they cannot be strictly defined by geography. There were no specific criteria for including patients in the study—it required that the study personnel be present, that the densitometry technologist had time to perform additional images, and that the subjects agreed to participate. When our convenience sample was compared with all adult patients (total of 10,547) who had BMD measured during the same time at the same densitometry facility, the study subjects were slightly older [62.6 ± 13.9 (SD) versus 60.0 ± 15.3 years, *p* < .0001], had a higher percentage of women (89% versus 86%, *p* = .003) and white patients (62% versus 49%, *p* < .001), and had a lower average femoral neck BMD (0.789 versus 0.861 g/cm^2^, *p* < .0001).

### Measurements

Each subject completed a questionnaire that included information on personal and family history of fractures and their circumstances, young-adult height and weight, history of medical problems, medication use, and personal habits such as smoking, alcohol consumption, calcium intake, and activity level. Height and weight were measured using standard clinic equipment. The 10-year probability of major osteoporotic fractures was calculated based on the FRAX algorithm using the Web-based calculator (http://www.shef.ac.uk/FRAX).

Vertebral fracture assessment (VFA) and BMD measurements of the lumbar spine and proximal femur were obtained by two International Society for Clinical Densitometry (ISCD)-certified technologists using the Prodigy densitometer (GE Medical Systems, Madison, WI, USA). The precision of BMD measurement was 1% for lumbar spine and total hip and 1.5% for the femoral neck. National Health and Nutrition Examination Survey (NHANES) III data were used to derive *T*-scores (gender-adjusted Caucasian norms) and *Z*-scores (age-, gender-, race-, and weight-adjusted norms). BMD of L1–4 with elimination of artifact-labeled vertebrae was used for lumbar spine, whereas the lower of left and right sides were used for femoral neck and total hip measurement.

All VFA images were evaluated by one ISCD-trained clinician (TJV) using the Genant semiquantitative (SQ) approach,(19) as recommended by the ISCD(20,21): Vertebrae that contain a fracture on visual inspection are assigned a grade such that grade 1 (mild) fracture represents a reduction in vertebral height of 20% to 25%, grade 2 (moderate) a reduction of 26% to 40%, and grade 3 (severe) a reduction of over 40%. When examining the association between RTA and the presence of prevalent vertebral fractures found on VFA, we calculated FRAX without entering VFA-identified fracture in the “history of fractures” field in FRAX calculator (unless they were self-reported in the questionnaire).

BMD and radiographic images of the left calcaneus were obtained in duplicate using the Peripheral Instantaneous X-Ray Imager (PIXI; GE Medical Systems, Madison, WI, USA) equipped with a high-resolution camera. The precision of the heel BMD measurements was 1.8%. For each subject, the mean of the two heel measurements was used for BMD and RTA values. PIXI has a 5-second acquisition time and provides 512 × 512 pixel high- and low-energy (80 and 55 kVp) 12-bit images with 200 µm pixels. It uses a gadolinium oxysulfide phosphor screen coupled with a CCD camera via a lens system. Low-energy images were used for RTA.

### Calculation of RTA features

**Fig. 1:**
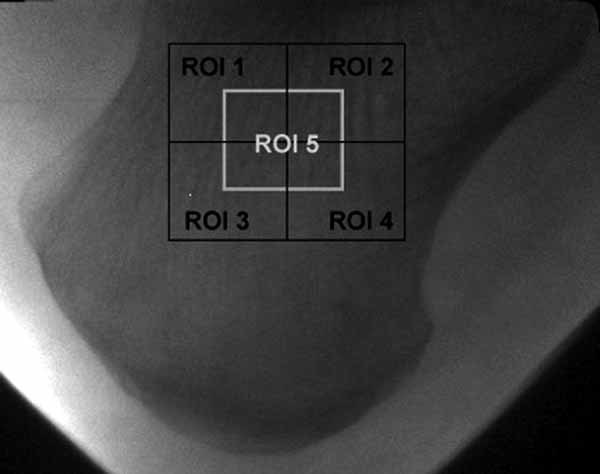
PIXI image with larger ROI (128 × 128 pixel) and a smaller central ROI used for RTA (ROI5 with 64 × 64 pixels).

RTA was performed as described previously.(6,7,13,16,18) On each calcaneal image, a trained operator placed a 128 × 128 pixel region of interest (ROI) that was then divided into five smaller (64 × 64 pixel) ROIs (Fig. 1). In previous studies, we found that the central ROI (ROI5 in Fig. 1) was best suited for RTA because it yielded the highest precision and the best separation between patients with and without vertebral fractures.(16,18) The location of the ROI for RTA is proximal to the area where BMD is measured because we and others have shown that the more proximal area has a richer trabecular structure.(22,23)

The details of RTA have been reported previously.(6,7,13,16,18) Briefly, the Fourier-based spectral analysis yields the root mean square (RMS) variation and the first moment of the power spectrum (FMP), as well as related directional measures sdRMS and minFMP. Prior to Fourier analysis, background trend correction using a second-order polynomial least-square fit is performed on each ROI. RMS, used to characterize the magnitude of the trabecular texture pattern, is a measure of the variability in the radiographic texture pattern. It is expressed in gray-scale level, with higher values corresponding to stronger bone. FMP characterizes spatial frequency in the radiographic pattern and is expressed in cycles per millimeter, with lower values corresponding to stronger bone. Because cancellous bone exhibits a preferential orientation of the trabeculae, the RTA can yield descriptors of directional dependence by dividing the power spectrum into 24 radial sectors at 15-degree intervals and performing the summations within each sector. These directional features include sdRMS (standard deviation of the RMS), which provides a measure of anisotropy, and minFMP (minimum FMP), which represents the lowest spatial frequency among the 24 segments. Stronger bone is expected to yield higher sdRMS and lower minFMP values.

Fourier analysis also can characterize the roughness/smoothness of the textural pattern from power law spectral analysis, which yields a feature beta,(24) with higher values corresponding to stronger bone. Beta is related to fractal dimension *D* through beta = 8–2*D*.

The precision of RTA features (coefficients of variation) was 0.77% for iRMS, 6.79% for sdRMS, 1.07% for iFMP, 3.38% for minFMP, and 5.56% for beta.

### Statistical analysis

Differences between subgroups of patients (such as male versus female or subjects with and without vertebral fractures) were examined using *t* tests for continuous and chi-square tests for categorical variables. The correlations between individual RTA features and between RTA and heel BMD were examined using Pearson correlation. The association between RTA and clinical variables was modeled using linear regression (with RTA features as the outcomes), whereas the association of RTA with prevalent vertebral fractures was modeled using multivariate logistic regression analysis with presence of vertebral fractures as a binary outcome. When performing these regression analyses, RTA results were standardized (expressed in standard deviations derived from the study population), which allowed us to compare the strength of the associations of RTA features and of BMD *T*-scores to clinical variables. All analyses were performed using the STATA 10 statistical package.

## Results

From the total of 1058 subjects who consented to participate, 900 were available for analysis. The remaining 158 subjects were excluded because heel images were not obtained, positioning of the heel was poor, the heel was too large to fit in the PIXI positioner, or heel imaging was performed on a different PIXI that was later found to have different imaging characteristics (higher RMS-based features owing to higher exposure).

The clinical characteristics of the 900 subjects included in analysis are shown in Table 1. Although BMD *T*-scores were not significantly different between the genders, men had lower *Z*-scores (*p* < .0001) and higher prevalence of glucocorticoid use and vertebral fractures (see Table 1).

**Table 1:** Clinical Characteristics of the Study Subjects Given as Mean ± SD for Continuous and Counts (%) for Categorical Variables

	All subjects (900)	Females (806)	Males (94)
Age (years)	62. 8 ± 13.9	63.0 ± 13.7	60.5 ± 15.2
Race			
African American	351 (33%)	275 (34%)	22 (24%)
Asian	38 (4%)	33 (4%)	3 (3%)
Caucasian	660 (61%)	480 (60%)	65 (69%)
Hispanic	26 (2%)	18 (2%)	4 (4%)
Weight (lb)*	154 ± 37	152 ± 36	176 ± 37
BMI	27 ± 6	27 ± 6	27 ± 5
Vertebral fractures*	190 (23%)	157 (21%)	33 (38%)
Peripheral fractures	221 (27%)	212 (28%)	19 (22%)
Glucocorticoid use*	176 (20%)	142 (18%)	34 (36%)
BMD *T*-score			
Lumbar spine	−1.6 ± 1.5	−1.6 ± 1.5	−1.7 ± 1.7
Femoral neck	−2.1 ± 1.1	−2.1 ± 1.1	−2.1 ± 1.1
Lowest hip or spine	−2.5 ± 1.2	−2.5 ± 1.2	−2.6 ± 1.2
Heel	−1.0 ± 1.4	−0.9 ± 1.4	−1.4 ± 1.6
BMD *Z*-score			
Lumbar spine*	−0.8 ± 1.0	−0.7 ± 1.4	−1.4 ± 1.6
Femoral neck*	−1.0 ± 1.0	−0.9 ± 1.0	−1.3 ± 1.0
Lowest hip or spine*	−1.4 ± 1.1	−1.4 ± 1.1	−1.9 ± 1.1
Heel*	−0.6 ± 1.4	−0.6 ± 0.1.3	−1.2 ± 1.6
FRAX (%)*	21 ± 16	21 ± 16	17 ± 10
Osteoporosis Tx	336 (37%)	306 (38%)	30 (32%)

*Note:* Vertebral fracture status was not available in 58 subjects who had uninterpretable or missing VFA; FRAX is reported as 10-year probability of sustaining major osteoporotic fracture. “Peripheral fractures” is a binary variable (yes or no) and refers to nonvertebral fragility fracture that occurred after age 50. “Glucocorticoid use” is binary variable with “yes” defined as cumulative exposure of at least 5 mg/day of prednisone or equivalent for at least 3 months.

* *p* < .001 for gender differences.

### Correlation between RTA features

This was significant for all RTA features (Table 2). RTA features also were correlated with heel BMD, but the correlations were low (*R*^2^ between 0.04 and 0.15). Some RTA features also were correlated with BMD at central sites, but the correlation coefficients were even lower than for the heel BMD (data not shown).

**Table 2:** Correlation Between Individual RTA Features and Heel BMD Presented as Correlation Coefficients (*R*)

	iRMS	sdRMS	iFMP	minFMP	Beta
sdRMs	0.84				
iFMP	−0.85	−0.87			
minFMP	−0.74	−0.88	0.91		
Beta	0.74	0.75	−0.79	−0.71	
BMD_heel_	−0.39	0.27	0.20	0.23	−0.22

*Note:* *p* < .0001 for all correlations.

### Relationship between RTA and anthropomorphic and clinical variables

This is shown in Table 3. The relationship of heel BMD to the same variables is presented for comparison. Heel BMD decreased with age and increased with weight and was lower in men than in women. Although heel BMD (*T*-score) was, on average, higher in African Americans, when adjusted for age, weight, and gender (*Z*-scores), it was lower by 0.3 *Z*-score units (*p* < .001).

**Table 3:** Association of Heel BMD and RTA Features with Anthropometric and Clinical Characteristics Derived from Multivariate Regression Analysis

	Heel BMD	iRMS	sdRMS	iFMP	minFMP	Beta
**Age/10 years**						
Coefficient	−**0.277**	−**0.115**	−**0.104**	**0.158**	**0.152**	−**0.117**
95% CI	−0.33, −0.21	−0.16, −0.07	−0.15, −0.6	0.11, 0.21	0.11, 0.20	−0.17, −0.07
*p* value	<0.001	<0.001	<0.001	<0.001	<0.001	<0.001
**Weight/10 lb**						
Coefficient	**0.191**	− **0.081**	−**0.082**	**0.109**	**0.088**	−**0.089**
95% CI	0.16, 0.21	−0.09, −0.06	−0.10, −0.06	0.09, 0.12	0.07, 0.10	−0.11, −0.07
*p* value	<0.001	<0.001	<0.001	<0.001	<0.001	<0.001
**Gender: male**						
Coefficient	−**0.870**	−**0.415**	−**0.250**	0.148	**0.212**	−**0.188**
95% CI	−1.13, −0.61	−0.61, −0.22	−0.46, −0.04	−0.05, 0.35	0.01, 0.42	−0.40, 0.02
*p* value	<0.001	<0.001	0.02	0.15	0.04	0.077
**Race: AA**						
Coefficient	**0.338**	−**0.276**	−**0.142**	0.066	0.068	−0.008
95% CI	0.16, 0.51	−0.40, −0.15	−0.28, −0.01	−0.06, 0.20	−0.06, 0.20	−0.14, 0.12
*p* value	<0.001	<0.001	0.036	0.30	0.309	0.90
**Heel T-score**						
Coefficient		−**0.166**	−**0.089**	0.022	**0.071**	−**0.054**
95% CI		−0.21, −0.12	−0.14, −0.04	−0.03, 0.07	0.02, 0.12	−0.10, −0.00
*p* value		<0.001	0.001	0.37	0.006	0.041
**GC use**						
Coefficient	− 0.201	−**0.223**	−**0.165**	**0.155**	**0.156**	−**0.219**
95% CI	−0.4, 0.001	−0.37, −0.08	−0.32, −0.09	0.003, 0.31	0.001, 0.31	−0.38, −0.06
*p* value	0.051	0.003	0.039	0.046	0.049	0.007
**Vertebral Fx**^a^						
Coefficient	−**0.311**	−**0.157**	−**0.224**	**0.198**	**0.162**	−**0.300**
95% CI	−0.52, −0.10	−0.30, −0.01	−0.37, −0.07	0.04, 0.34	0.01, 0.31	−0.45, −0.14
*p* value	0.004	0.03	0.004	0.009	0.036	<0.001

*Note:* Results are expressed per standard deviation of each RTA feature to facilitate the comparison between the RTA and heel BMD *T*-scores. Coefficients with the significance of the effect with *p* < .05 are in boldface.

a Association between RTA features and vertebral fractures (while controlling for all other predictors form the table) was examined in 842 patients who had information available on vertebral fractures.

Among RTA features, more osteoporotic, weaker bone is associated with lower RMS and beta and higher FMP values. This combination of RTA findings was associated with increasing age. However, RMS and beta also were lower and FMP higher with increasing weight and BMD *T*-score. Similar to heel BMD, RTA features suggested more washed-out trabecular structure among males compared with females and among African Americans compared with Caucasians. When controlling for the predictors listed in Table 3, no significant association was found between RTA and smoking, years since menopause, exercise, and use of calcium supplements or pharmacologic therapies for osteoporosis (data not shown).

### Relationship between RTA and fragility

*Fragility* was defined as presence of vertebral fractures. Information on vertebral fractures was available in 842 of 900 subjects. The remaining subjects were excluded from this analysis because their spine image was missing or could not be interpreted owing to scoliosis or degenerative changes. When controlling for other predictors from Table 3, all RTA features had a significant association with vertebral fractures, with the strongest association observed for beta. In the analyses below, we used femoral neck *T*-score because this BMD measurement is used in the FRAX calculation. Similar results were obtained when total hip or lowest of hip or spine *T*-score was used in the analyses instead of femoral neck, although the prevalence odds ratio (POR) was slightly lower than for the femoral neck *T*-score (data not shown).

Among the 784 subjects who were over age 40, which permitted calculation of FRAX, and on whom we had information on vertebral fractures, we found a significant association between prevalent vertebral fractures and FRAX (Table 4*A*), with odds of having prevalent vertebral fractures (POR) of 1.6 [95% confidence interval (CI) 1.7–1.8] per 10% increase in 10-year probability of major osteoporotic fracture.

**Table 4:** Logistic Regression Models Predicting Prevalent Vertebral Fractures (VFx) Relative to (*A*) FRAX and/or Individual Risk Factors and (*B*) RTA Feature Beta Combined with FRAX or Individual Risk Factors

	All (*N* = 784, 23% VFx)			Women (*N* = 707, 21% VFx)			Men (*N* = 77, 40% VFx)		
			
Predictor(s)	POR^a^	95% CI	*p* Value	POR^a^	95% CI	*p* Value	POR^a^	95% CI	*p* Value
A. FRAX alone (1), FRAX with risk factors (2), or risk factors alone (3)									
(1) FRAX	**1.6**	1.4, 1.7	<.001	**1.6**	1.4, 1.8	<.001	**2.0**	1.0, 3.7	0.025
(2) FRAX + risk factors									
FRAX	1.0	0.8, 1.2	.6	1.1	0.9, 1.4	.242	1.0	0.3, 2.8	.982
T-score	**1.6**	1.2, 2.0	.001	**1.3**	1.0, 1.7	.04	**3.9**	1.6, 9.7	.004
Age/decade	**1.5**	1.2, 1.8	<.001	**1.7**	1.4, 2.2	<.001	0.7	0.4, 1.2	.251
Glucocorticoid use	**2.0**	1.3, 3.3	.003	**2.0**	1.2, 3.5	.013	1.0	0.2, 3.7	.986
Peripheral fracture	1.6	1.0, 2.6	.044	1.6	1.0, 2.7	.055	1.3	0.2, 7.0	.779
(3) Risk factors									
T-score	**1.6**	1.3, 2.0	<.001	**1.5**	1.2, 1.8	<.001	**3.9**	1.8, 8.4	<.001
Age/decade	**1.6**	1.3, 1.9	<.001	**1.8**	1.5, 2.2	<.001	0.7	0.4, 1.2	.235
Glucocorticoid use	**2.1**	1.4, 3.3	.001	**2.2**	1.3, 3.6	.002	1.0	0.3, 3.0	.972
Peripheral fracture	**1.7**	1.1, 2.6	.014	**1.9**	1.2, 2.9	.005	1.3	0.3, 6.0	.749
B. RTA feature beta alone (1), combined with FRAX (2), or individual risk factors (3)									
(1) Beta	**1.5**	1.2, 1.8	<.001	**1.5**	1.2, 1.8	<.001	1.2	0.7, 1.8	.55
(2) Beta + FRAX									
Beta	**1.5**	1.2, 1.8	<.001	**1.5**	1.2, 1.9	<.001	1.0	0.6, 1.6	.961
FRAX	**1.6**	1.4, 1.7	<.001	**1.6**	1.4, 1.8	<.001	**1.9**	1.1, 3.7	.03
(3) Beta + risk factors									
Beta	**1.5**	1.2, 1.8	<.001	**1.5**	1.2, 1.8	.001	1.3	0.7, 2.2	.277
T-score	**1.7**	1.4, 2.1	<.001	**1.6**	1.2, 1.9	<.001	**4.2**	1.8, 9.1	<.001
Age/decade	**1.5**	1.2, 1.8	<.001	**1.8**	1.4, 2.2	<.001	0.7	0.4, 1.1	.216
Glucocorticoid use	**1.8**	1.2, 2.9	.008	**1.9**	1.2, 3.2	.011	0.9	0.2, 2.7	.856
Peripheral fracture	**1.6**	1.1, 2.5	.026	**1.8**	1.2, 2.9	.008	1.1	0.2, 5.4	.904

a POR, prevalence odds ratio = odds of having a prevalent vertebral fracture on VFA. For FRAX, POR is expressed per 10% increase in the 10-year absolute risk of major osteoporotic fracture; for BMD *T*-score, per 1 unit *decrease* in femoral neck BMD; for age. per 1 decade *increase;* and for RTA, per 1 standard deviation *decrease* in beta. POR values with the significance of the effect with *p* < .05 are in boldface. For definition of “peripheral fracture” and “glucocorticoid use,” see Table 1.

Although RTA features were not correlated with FRAX in univariate analysis, after adjusting for weight, most RTA features showed a statistically significant (*p* = .001) correlation with FRAX score, although the slope was relatively shallow (only 0.1 standard deviation or less change in RTA per 10% increase in 10-year probability of fracture).

As seen in Table 3, among the RTA features, Beta had the most significant association with vertebral fractures and therefore was selected for inclusion in the multivariate models shown in Table 4*B*. Beta was significantly associated with prevalent vertebral fractures alone (Table 4*B*, model 1) or when controlling for FRAX (Table 4*B*, model 2) or individual risk factors (Table 4*B*, model 3). This was certainly the case in women (Table 4*B*, middle column), in whom the odds of having vertebral fractures increased by 50% per standard deviation decrease in beta whether used alone or controlled for FRAX or individual risk factors. In the much smaller study population of men, after controlling for individual risk factors (Table 4*B*, model 3, last column), the POR for the effect of beta on odds of having vertebral fractures was similar to that seen in women, although it was not statistically significant. RMS- and FMP-based features also were associated with vertebral fractures alone or when controlling for FRAX or individual risk factors, although the POR was lower but still significant (POR of 1.3 to 1.4, *p* = .001 to .003). Controlling for heel BMD did not change the association between beta and vertebral fractures. This was true if heel BMD was added to any of the models in Table 4*B* or if heel *T*-score was used in model 3 in Table 4*B* instead of femoral neck *T*-score.

### Effect of osteoporosis treatment on RTA and its association with vertebral fractures

Use of pharmacologic therapy for osteoporosis did not have a significant association with RTA when added to the multivariate models presented in Table 3. Similarly, there was no significant association between vertebral fractures and treatment or treatment-RTA interaction when added to the models shown in Table 4. However, the association of vertebral fractures with beta as well as with FRAX was stronger in 297 treated than in 485 untreated patients, although the confidence intervals overlapped. PORs (95% CI) in treated versus untreated subjects were for beta 1.6 (1.2–2.2, *p* = .003) versus 1.4 (1.1–1.8, *p* = .004) and for FRAX 1.8 (1.5–2.2, *p* < .001) versus 1.4 (1.2–1.6, *p* < .001).

## Discussion

Our results indicate that clinically useful information about fracture risk can be obtained using RTA, which differentiated subjects with and without vertebral fractures even when controlling for BMD and clinical risk factors (see Table 4). This is observed when the latter are included in the model as BMD, age, history of glucocorticoid use, and prior fragility fracture or combined into a summary measure such as FRAX. When added to such models, RTA feature beta had a significant effect with an odds ratio of having a vertebral fracture of 1.5 per 1 standard deviation decrease in beta. These findings suggest that as a relatively simple, economic method for assessing bone structure, RTA may provide additional information about bone fragility not captured by BMD measurement and clinical risk factors. It should be noted that the RTA measurements are not just another way of assessing BMD. This is supported by finding relatively low (albeit statistically significant) correlation between RTA features and heel BMD (see Table 2) and by observing that adding heel BMD did not affect the association between vertebral fractures and RTA feature beta shown in Table 4.

Our study is unique in that it examined the performance of RTA not in a case-control study but in a cross-sectional evaluation of patients referred for bone densitometry as part of their routine clinical care. Previous case-control studies have shown differences in RTA between subjects with clinically diagnosed osteoporotic fractures and healthy age-matched nonfracture controls.(8–10,25) In contrast, we recruited patients referred for densitometry and compared subjects with and without vertebral fractures newly found on the VFA. Consequently, our subjects without fractures were at least suspected of having increased bone fragility that prompted their referral for BMD testing, whereas subjects with VFA-detected vertebral fractures were likely to be less fragile than those with clinical osteoporotic fractures. Therefore, the differences in bone fragility between patients with and without vertebral fractures in our study are likely to be smaller than the differences between fracture patients and healthy controls in previous studies.(6–10,25) Nevertheless, we found significant differences in RTA between densitometry patients with and without (previously undetected) vertebral fractures, arguably an area where further stratification of fracture risk would have the greatest benefit. Furthermore, because our study included subjects of different ages, genders, and races, as well as treated and untreated subjects, our results are broadly applicable to the general densitometry population.

We also examined the relationship between RTA and clinical variables. As expected, we found that increasing age was associated with the RTA pattern seen in washed-out fragile bone, i.e., lower RMS and beta and higher FMP, similar to what has been reported for calcaneal radiographs.(25) However, we found that the same pattern of RTA features also was associated with increasing weight and heel BMD. This seemingly paradoxical finding is explained by the observation that a higher amount of fat in the bone marrow produces a more washed-out appearance of the radiographic texture pattern.(26) Similarly, a larger heel, which also has a higher BMD, will appear relatively washed out on a densitometric image because the X-ray exposure is held constant in the PIXI system, resulting in a noisier image of a thick object owing to photon attenuation.

Another interesting observation from our study is that the males were more fragile than the females, as manifested by higher prevalence of vertebral fractures and lower BMD scores. This is in contrast to population studies, where males typically have lower prevalence of osteoporosis and lower fracture rates. However, the prevalence of vertebral fractures in our male subjects is similar to that reported in men referred for bone density testing,(27) suggesting that males undergoing BMD testing are likely to be more “osteoporotic” than females, presumably because males are referred when they have diseases or use medication that cause osteoporosis, whereas women may undergo BMD testing as routine screening in the absence of specific risk factors. This is also a likely explanation for lower BMD and worse (more washed-out) texture pattern in males in our study (see Table 3). Similarly, lower BMD and lower RMS-based RTA features observed in African-American subjects in our study may also be due to selection bias, with African Americans being referred for densitometry when they are suspected of having significant pathology rather than for routine screening.

Although not an initial goal of our study, our data provided an opportunity to examine gender differences in the relationship between prevalent vertebral fractures and FRAX in subjects undergoing bone densitometry. Because vertebral fractures are a strong predictor of future fractures,(28–30) they are often used as a marker of fragility. In women (see Table 4*A*, middle column), FRAX adequately captured the association between individual predictors and vertebral fractures, except for age and history of glucocorticoid use, which remained significant when controlling for FRAX. This may be due to a stronger association of these predictors with vertebral fractures than with major osteoporotic fractures, for which FRAX was developed. A different picture emerged in men (see Table 4*A*, right column): The only significant predictor of vertebral fractures was BMD *T*-score, which was not affected by adding FRAX or other clinical risk factors. One could argue that the results in men are distorted because men have a higher prevalence of traumatic vertebral fractures. However, this is unlikely because inclusion of traumatic fractures would decrease rather than increase the strength of association between BMD and vertebral fractures. The reason for the unusually high POR for the association of prevalent vertebral fractures and BMD in our male subjects is not clear, although similarly high odds ratio have been found for an association of hip fractures and femoral neck *T*-score in the Osteoporotic Fractures in Men Study (MrOS).(31) Further studies are needed to determine whether applicability of FRAX to patients undergoing densitometry differs by gender.

Another interesting observation was that the strength of association between the RTA feature beta and vertebral fractures was, if anything, stronger in subjects who were receiving pharmacologic therapy for osteoporosis. Since the same is true for FRAX, the likely explanation is that treated subjects were more “osteoporotic,” which is confirmed by significantly lower *T*-scores in the treated as compared with untreated subjects (−2.7 versus −2.4, *p* = .002), and thus more likely to show the differences in bone structure associated with fragility.

There are limitations to our study. We did not have a sufficient number of males to draw firm conclusions regarding gender differences in RTA or the association of RTA and fragility. However, we found no significant interaction between RTA and gender with regard to vertebral fractures, suggesting that RTA had similar predictive value in both men and women. Another limitation is the inclusion of both treated and untreated subjects. However, we found no significant effect of treatment in the multivariate analyses in Table 4 and no interaction between treatment and RTA. Further, as mentioned earlier, we observed that the association between vertebral fractures and the RTA feature beta was stronger in treated patients. On the other hand, inclusion of treated subjects makes our results more applicable to the broad range of subjects referred for bone densitometry, many of whom are currently receiving treatment for osteoporosis.

Another possible limitation is that we used a convenience sample and did not include all patients referred for densitometry. Because many of our study subjects are clinic patients of the first author (TJV), who has an osteoporosis referral practice, they are likely to be more “osteoporotic” than the general densitometry population, an assumption confirmed by higher age and lower BMD of the study population compared with all subjects referred for BMD testing to our facility during the same time period (see Methods). Arguably, however, this relatively high-risk patient population is the group where a further refinement of fracture risk would be most useful. Finally, PIXI densitometer is no longer produced commercially, and in addition, the instrument we used was not an ordinary PIXI but was especially equipped with a high-resolution camera. However, our findings are a proof of concept and can be applied to either another commercially available system (described in refs. (25) and (32)) or can lead to the development of new technology aimed at combining assessment of both bone mass and bone structure using a portable system.

In conclusion, we found that RTA performed on densitometric calcaneal images had a significant association with prevalent vertebral fractures even when controlling for clinical risk factors and BMD. This suggests that RTA provides additional information about bone fragility not captured by currently used predictors.
